# Effectiveness of Tolvaptan for Autosomal Dominant Polycystic Kidney Disease in US Clinical Practice With Comparison to Historical Control Data

**DOI:** 10.1016/j.xkme.2025.100988

**Published:** 2025-02-27

**Authors:** Ronald D. Perrone, Diana Garbinsky, Sasikiran Nunna, Hema K. Gandhi, Ancilla W. Fernandes, Gabriela Burgos, Abisola Olopoenia, Marc DeCongelio, Martine C. Maculaitis, Xiaolei Zhou

**Affiliations:** 1 Division of Nephrology, Tufts Medical Center, Boston, MA; 2 RTI Health Solutions, Research Triangle Park, NC; 3 Otsuka Pharmaceutical Development & Commercialization Inc, Rockville, MD; 4 Oracle Life Sciences, Austin, TX

**Keywords:** Autosomal dominant polycystic kidney disease, tolvaptan, clinical practice, historical control study, estimated glomerular filtration rate

## Abstract

**Rationale & Objective:**

Data from clinical practice are needed to characterize the effectiveness of pharmacotherapy outside the controlled setting of clinical trials but lack an untreated placebo group for comparison. To assess the effectiveness of tolvaptan for autosomal dominant polycystic kidney disease (ADPKD) in nephrology practice, we performed a chart review of US patients and compared it with a historical matched control cohort.

**Study Design:**

Patient data from charts were provided by US nephrologists who participated in an online survey. Historical control data for patients with ADPKD not treated with tolvaptan were extracted from a database of ADPKD clinical studies (Consortium for Radiologic Imaging Studies of Polycystic Kidney Disease, HALT Progression of Polycystic Kidney Disease, and OVERTURE).

**Setting & Participants:**

Nephrologist respondents (n = 57) provided baseline data and up to 4 years of follow-up on estimated glomerular filtration rate (eGFR) for tolvaptan-treated adults (n = 149). Historical ADPKD-affected controls were adults in Mayo imaging risk classes 1C–1E (ie, at increased risk of rapid progression, consistent with the tolvaptan indication).

**Exposure:**

Cases had to receive tolvaptan continuously for ≥2 years. Historical controls received nontolvaptan standard of care, including various antihypertensive regimens.

**Outcome:**

Annual rate of eGFR change.

**Analytical Approach:**

Cases and controls were matched on baseline clinical characteristics (matched set A: age, sex, and chronic kidney disease stage [110 matched pairs]; matched set B: age, sex, and eGFR [98 matched pairs]) and compared using a mixed model.

**Results:**

The annual rate of eGFR decline was slower in tolvaptan-treated patients versus historical controls, by 1.40 mL/min/1.73 m^2^ (95% CI, 0.05-2.74; *P* = 0.04) in set A. Set B demonstrated a similar trend: 1.18 mL/min/1.73 m^2^ per year (95% CI, −0.22 to 2.58; *P* = 0.10).

**Limitations:**

Risk of bias from convenience sampling and potential residual confounding after case/historical control matching.

**Conclusions:**

Tolvaptan was associated with slower eGFR decline in routine clinical practice, consistent with the results of controlled trials.

## Plain Language Summary

Randomized, controlled trials are the gold standard for evaluating pharmacotherapy, but evidence collected under routine clinical conditions can answer questions about drug effectiveness, safety, and value that are particularly relevant to everyday medical practice. Trial data have demonstrated that tolvaptan slows kidney function decline in patients with autosomal dominant polycystic kidney disease who are at increased risk of rapid progression. To assess treatment effect in regular nephrology practice, we invited US nephrologists to provide anonymized data on patients receiving tolvaptan under their care. We compared decline in estimated glomerular filtration rate in the tolvaptan-treated group with matched historical controls who did not receive tolvaptan. Across multiple matched analysis sets, tolvaptan slowed estimated glomerular filtration rate decline over a 4-year follow-up period.

Tolvaptan has demonstrated efficacy in decreasing the rate of kidney function decline among patients with autosomal dominant polycystic kidney disease (ADPKD) who are at elevated risk of rapid progression. The evidence is robust, including large-scale (participant n > 1,000), randomized, placebo-controlled clinical trials with 1-3 years of follow-up (TEMPO 3:4 and REPRISE) and long-term, open-label extension data (TEMPO 4:4).^1^, ^2^, ^3^ Although randomized, controlled clinical trials are the gold standard for evidence generation, other methodologies are needed to answer questions specific to the effectiveness, safety, and value of pharmacotherapy in everyday medical practice. Evidence collected via electronic health records, claims and billing data, patient registries, surveys, and mobile health applications enables assessment in routine clinical contexts.^4^^,^^5^

Recently, a postmarketing surveillance study of Japanese patients with ADPKD who were treated with tolvaptan in regular clinical settings (NCT02847624) demonstrated that tolvaptan was effective in reducing the rate of estimated glomerular filtration rate (eGFR) decline relative to the period before treatment.^6^ Data on the effectiveness of tolvaptan in everyday practice are needed for other populations. This need is especially relevant, given that tolvaptan is the only disease-modifying treatment available for patients at risk of rapid progression, with other interventions in ADPKD limited to nonspecific measures such as blood pressure control and lifestyle management.^7^ We therefore invited US nephrologists to provide data from patient charts for the purpose of evaluating the effects of tolvaptan on the annual rate of change in kidney function. To compare tolvaptan treatment with no tolvaptan treatment, we used a research design that included historical controls with ADPKD who did not receive tolvaptan, and we matched cases and controls to minimize the effects of any differences between the compared groups.

## Methods

### Data Collection and Eligibility Criteria

#### Tolvaptan-Treated Cohort (Cases)

To obtain clinical practice data on patients treated with tolvaptan for ADPKD, an online survey of US nephrologists was conducted from May 20, 2022, to September 19, 2022. A convenience sample of physicians was recruited by Cerner Enviza and its panel partners. Nephrologists from practices of all types across the United States were invited to participate through an email that contained a link to the survey. To qualify for the survey, nephrologists had to meet the following criteria: practiced nephrology for ≥2 years and prescribed tolvaptan for the treatment of ADPKD to ≥1 adult patient for whom complete medical records were available. An honorarium of US$156 was provided for participation.

Nephrologist participants provided the charts of patients who met the following inclusion criteria: aged 18 years or older; diagnosed with ADPKD; and treated continuously with tolvaptan for ≥2 years (ie, no interruptions longer than 60 days). Exclusion criteria were: history of dialysis, kidney transplantation, or kidney malignancy; previous participation in a tolvaptan clinical trial; or previous treatment with tolvaptan for hyponatremia. To help minimize selection bias, the nephrologists were asked to provide, whenever possible, data on 3 consecutive patients who were treated with tolvaptan, starting with the first patient treated with tolvaptan after April 2018, regardless of the outcomes observed.

#### Non–Tolvaptan-Treated Historical Cohort (Controls)

Data on patients with ADPKD who were not treated with tolvaptan were obtained in part from studies sponsored by the National Institutes of Health and available in the National Institute of Diabetes and Digestive and Kidney Diseases Central Repository. These were the Consortium for Radiologic Imaging Studies of Polycystic Kidney Disease (CRISP) I and II (2001-2012; NCT01039987) and HALT Progression of Polycystic Kidney Disease (HALT-PKD) Study A (2006-2014; NCT00283686).^8^^,^^9^ CRISP is a long-term natural history study, and HALT-PKD evaluated various antihypertensive regimens in ADPKD. The other source study was OVERTURE (NCT01430494), an Otsuka-sponsored, longitudinal, observational study of patients with ADPKD treated with standard of care that was conducted before commercial availability of tolvaptan for ADPKD in the United States.^10^

Patient eligibility criteria were designed to select a population of US adults who were at increased risk of rapid ADPKD progression, consistent with the indication for tolvaptan therapy. Inclusion criteria were: US patients aged 18 years or older, Mayo imaging risk classes 1C, 1D, or 1E (increased risk of rapid progression).^11^ Exclusion criteria were: Mayo imaging risk classes 1A or 1B (low risk), treated with tolvaptan in CRISP, or randomized to low-blood-pressure control arms in HALT-PKD Study A. Total kidney volume for determination of risk class was assessed by magnetic resonance imaging in all studies.

### Outcomes

Outcomes evaluated were eGFR over time and annual rate of change in eGFR. In the tolvaptan cohort, eGFR and serum creatinine were reported at month 1 and every 3 months from month 3 to month 48 in the chart review. When serum creatinine was known, eGFR was calculated based on serum creatinine, age, sex, and race using the Chronic Kidney Disease Epidemiology Collaboration 2009 equation.^12^ When serum creatinine was not available (<4% of patients), eGFR values reported in patient charts were used. Because the chart review did not capture eGFR at tolvaptan initiation (ie, baseline), the eGFR value at month 1 served as the baseline value for patients in the tolvaptan cohort and was used for matching in set B. For the historical control cohort, eGFR values were obtained from the database (mostly every 6 months), with the exclusion of eGFR assessments made after 4.5 years or collected after surgical or invasive radiologic procedures.

### Construction of the Matched Cohorts

Cases and controls were matched 1:1 for baseline clinical characteristics to minimize confounding in the comparison. Patients in the case and historical control cohorts were eligible for matching if they had ≥1 valid eGFR assessment during day 8 to year 1.5 and during year >1.5 to year 4.5. This was to ensure that patients had at least 2 data points during the follow-up. In addition, because tolvaptan was available for ADPKD for only ∼4 years in the United States at the time of chart review, data after 4.5 years (available in the control cohort) were excluded.

Eligible cases and controls were matched using the %GMATCH SAS macro developed by the Mayo Clinic,^13^ which uses a greedy matching algorithm, to create 2 matched analysis sets:

o Set A: age (±2 years), sex (female or male), chronic kidney disease (CKD) stage (G1, G2, G3a, G3b, G4, and G5).
o Set B: age (±2 years), sex (female or male), eGFR (±5 mL/min/1.73 m^2^).

The chart review captured baseline CKD stage (ie, at tolvaptan initiation), but not eGFR. Instead, eGFR at month 1 after tolvaptan initiation was collected. Although eGFR would provide more accurate matching, the month 1 eGFR is expected to be lower than the pretreatment eGFR because of the hemodynamic effect of tolvaptan (an acute drop in eGFR in the first week after treatment initiation that is reversible on discontinuation).^14^ Therefore, 2 sets of matching were conducted, one by baseline CKD stage and one by month 1 eGFR.

### Statistical Analyses

Based on statistical power calculations and feasibility assessment, a sample size of up to 75 nephrologists was targeted on the assumption that each physician would provide 2 to 3 charts, collecting data from up to ∼200 patients with ADPKD taking tolvaptan. Baseline demographic and disease characteristics were summarized for the full analysis set of cases and controls and each matched set.

Kidney function decline was compared between cases and controls in each matched analysis set using a mixed model with eGFR as the response variable. The model was used to estimate eGFR and change from baseline (theoretical baseline estimated from the mixed model) in eGFR at months 1, 12, 24, 36, and 48. The model included treatment, time (as a continuous variable), and a treatment-by-time interaction as fixed effects and patient-specific intercepts and slopes (for time) as random effects, which were assumed to have an unstructured covariance matrix. The Kenward-Roger approximation was used to estimate denominator degrees of freedom.^15^

### Ethical Conduct

The online survey received an exemption determination from Pearl IRB (Indianapolis, IN) on April 11, 2022 (protocol number 22-CERN-115). It was determined that this research posed no greater than minimal risk to the participants.

Privacy and confidentiality were respected in accordance with the applicable regulatory requirements. All study participants were anonymized (no direct or indirect identifiers), so neither the participants nor patient records could be identified. Accordingly, informed consent was not required.

## Results

### Analysis Sets

A total of 57 nephrologists with a mean (standard deviation [SD]) of 15.6 ± 7.4 years practicing medicine in nephrology completed the online survey, providing data on 149 patients. The year of tolvaptan initiation was 2018 for 21 patients (14%), 2019 for 72 patients (48%), and 2020 for 56 patients (38%). The mean ± SD duration of tolvaptan treatment was 2.7 ± 0.6 years, with a median of 2.6 years (range, 2.0-4.1 years).

**Figure 1:**
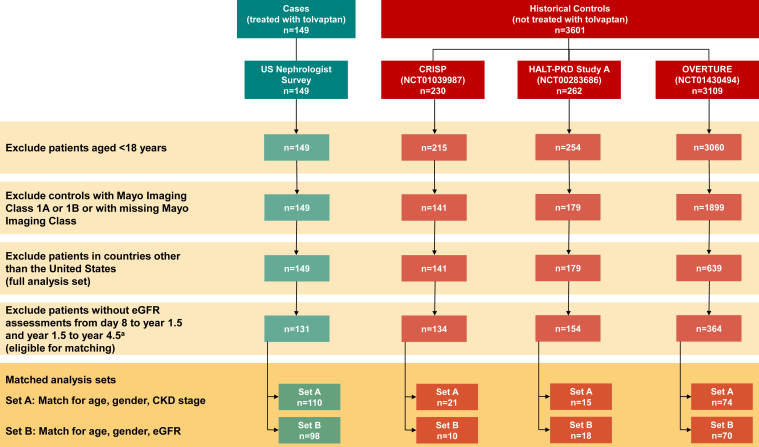
Summary of the cohort selection process for tolvaptan-treated cases and historical controls. CKD, chronic kidney disease; eGFR, estimated glomerular filtration rate. ^a^In the historical control cohort, eGFR assessments >4.5 years, eGFR assessments from the HALT-PKD low-blood-pressure control group for patients initiating in CRISP, and eGFR assessments collected after surgical or invasive radiological procedures were excluded.

Fig 1 summarizes the cohort selection process. The full analysis set consisted of 149 cases and 959 controls, of which 131 cases and 652 controls were eligible for matching. Of the patients receiving tolvaptan who were eligible for matching, 84% (n = 110) were matched with a historical control in matched analysis set A, and 75% (n = 98) were matched with a historical control in matched analysis set B.

### Patient Characteristics

The baseline characteristics of the tolvaptan-treated patients in the full analysis set reflected a wide spectrum of patient ages and disease progression stages, the latter indicated by baseline eGFR and CKD stage distribution (Table 1). No patient was in CKD stage G5 at baseline, consistent with the labeling for tolvaptan.^16^ Patient age at baseline ranged from 19-77 years, with a mean ± SD of 43 ± 12 years. The daily tolvaptan doses taken most recently by patients in the full analysis set spanned the dose range for maintenance treatment specified in the tolvaptan labeling (ie, 30 mg/d to 120 mg/d), which allows for titration based on tolerability or use of concomitant medications.^16^ The median tolvaptan dose was 60 mg/d (interquartile range, 30 mg/d).

**Table 1:** Patient Baseline Characteristics by Treatment Cohort in the Full Analysis Set

Characteristic	Tolvaptan (n = 149)	Historical Controls (n = 959)	Standardized Mean Difference
Age (y), n	149	959	
Mean ± SD	43.1 ± 11.7	42.5 ± 12.0	0.05
Range	19.4-77.0	18.1-70.9	
Sex, n (%)	149	959	
Female	60 (40.3)	494 (51.5)	−0.23
Race/ethnicity, n (%)	149	957	
White	93 (62.4)	814 (85.1)	−0.53
African American	22 (14.8)	48 (5.0)	0.33
Hispanic	12 (8.1)	61 (6.4)	0.06
Asian	12 (8.1)	15 (1.6)	0.31
Other	10 (6.7)	19 (2.0)	0.23
Age at ADPKD diagnosis (y), n	149	957	
Mean ± SD	35.0 ± 12.7	30.3 ± 12.6	0.36
Range	2.7-77.0	0.0-68.3	
CKD stage,^a^ n (%)	148	947	
G1	15 (10.1)	263 (27.8)	−0.46
G2	38 (25.7)	301 (31.8)	−0.14
G3a	53 (35.8)	129 (13.6)	0.53
G3b	22 (14.9)	117 (12.4)	0.07
G4	20 (13.5)	104 (11.0)	0.08
G5	0 (0.0)	33 (3.5)	−0.27
Baseline eGFR^b^ in mL/min/1.73 m^2^, n	148	947	
Mean ± SD	56.3 ± 26.7	68.6 ± 31.9	−0.42
Range	8.5-142.1	4.3-146.6	
History of nephrolithiasis, n (%)	149	959	
Yes	14 (9.4)	80 (8.3)	0.04
History of hypertension, n (%)	149	959	
Yes	104 (69.8)	819 (85.4)	−0.38

Abbreviations: ADPKD, autosomal dominant polycystic kidney disease; CKD, chronic kidney disease; eGFR, estimated glomerular filtration rate; SD, standard deviation.

a Stage G1, ≥90 mL/min/1.73 m^2^; stage G2, 60 to < 90 mL/min/1.73 m^2^; stage G3a, 45 to < 60 mL/min/1.73 m^2^; stage G3b, 30 to < 45 mL/min/1.73 m^2^; stage G4, 15 to < 30 mL/min/1.73 m^2^; stage G5, < 15 mL/min/1.73 m^2^.

b Because there were no baseline eGFR assessments for the tolvaptan patients, eGFR measured at month 1 (30-60 days after tolvaptan initiation) was considered as the baseline value.

The distribution of baseline characteristics in the tolvaptan cohorts of the 2 matched sets (Table 2) was generally similar compared with all eligible patients receiving tolvaptan. An exception was that the matched sets had slightly smaller proportions of tolvaptan-treated patients in CKD stage G4 (7% in sets A and B vs 14% in the full set). In each matched analysis set, age, sex, and eGFR were well-balanced between the tolvaptan and control cohorts (absolute standardized mean difference <0.2), confirming the success of the matching procedure.

**Table 2:** Patient Baseline Characteristics by Treatment Cohort in the Matched Analysis Sets

Characteristic	Matched Set A			Matched Set B		
	Tolvaptan (n = 110)	Historical Controls (n = 110)	Standardized Mean Difference	Tolvaptan (n = 98)	Historical Controls (n = 98)	Standardized Mean Difference
Age (y), n	110	110		98	98	
Mean ± SD	42.8 ± 10.1	42.9 ± 10.1	−0.01	43.5 ± 9.1	43.5 ± 9.1	−0.00
Range	19.4-69.8	19.3-69.6		23.2-67.3	21.9-67.0	
Sex, n (%)	110	110		98	98	
Female	44 (40.0)	44 (40.0)	0.00	37 (37.8)	37 (37.8)	0.00
Race/ethnicity, n (%)	110	110		98	98	
White	72 (65.5)	98 (89.1)	−0.59	69 (70.4)	85 (86.7)	−0.41
African American	16 (14.5)	4 (3.6)	0.39	12 (12.2)	4 (4.1)	0.30
Hispanic	8 (7.3)	4 (3.6)	0.16	6 (6.1)	5 (5.1)	0.04
Asian	7 (6.4)	2 (1.8)	0.23	4 (4.1)	1 (1.0)	0.20
Other	7 (6.4)	2 (1.8)	0.23	7 (7.1)	3 (3.1)	0.19
Age at ADPKD diagnosis (y), n	110	110		98	98	
Mean ± SD	35.0 ± 10.8	30.8 ± 11.2	0.38	35.6 ± 10.1	31.5 ± 10.8	0.40
Range	3.9-63.1	8.1-62.7		14.0-62.6	8.2-62.0	
CKD stage,^a^ n (%)	110	110		97	98	
G1	14 (12.7)	14 (12.7)	0.00	10 (10.3)	10 (10.2)	0.00
G2	34 (30.9)	34 (30.9)	0.00	27 (27.8)	35 (35.7)	−0.17
G3a	36 (32.7)	36 (32.7)	0.00	36 (37.1)	20 (20.4)	0.38
G3b	18 (16.4)	18 (16.4)	0.00	17 (17.5)	25 (25.5)	−0.20
G4	8 (7.3)	8 (7.3)	0.00	7 (7.2)	7 (7.1)	0.00
G5	0 (0.0)	0 (0.0)	n/a	0 (0.0)	1 (1.0)	−0.14
Baseline eGFR^b^ in mL/min/1.73 m^2^, n	110	110		98	98	
Mean ± SD	60.4 ± 26.4	63.2 ± 25.2	−0.11	57.9 ± 22.8	57.9 ± 22.4	0.00
Range	9.9-142.1	20.3-131.2		9.9-121.4	12.8-122.0	
History of nephrolithiasis, n (%)	110	110		98	98	
Yes	12 (10.9)	11 (10.0)	0.03	12 (12.2)	10 (10.2)	0.06
History of hypertension, n (%)	110	110		98	98	
Yes	79 (71.8)	99 (90.0)	−0.48	76 (77.6)	89 (90.8)	−0.37

Abbreviations: ADPKD, autosomal dominant polycystic kidney disease; CKD, chronic kidney disease; eGFR, estimated glomerular filtration rate; SD, standard deviation.

a Stage G1, ≥90 mL/min/1.73 m^2^; stage G2, 60 to < 90 mL/min/1.73 m^2^; stage G3a, 45 to < 60 mL/min/1.73 m^2^; stage G3b, 30 to < 45 mL/min/1.73 m^2^; stage G4, 15 to < 30 mL/min/1.73 m^2^; stage G5, < 15 mL/min/1.73 m^2^.

b Because there were no baseline eGFR assessments for the tolvaptan patients, eGFR measured at month 1 (30-60 days after tolvaptan initiation) was considered as the baseline value.

In matched set B, fewer patients in the control cohort than the tolvaptan cohort were in CKD stage G3a, even though their eGFR was matched within ±5 mL/min/1.73 m^2^. This difference was likely because of the fact that eGFR at month 1 was used as an approximation of the baseline value in the tolvaptan cohort. Also, small differences in eGFR within the ±5 mL/min/1.73 m^2^ could affect the classification to CKD stage G3a or G3b. Differences between treatment groups in the full analysis set were carried through to the matched sets: patients in the tolvaptan cohorts comprised a smaller proportion who self-identified as White than matched controls, and smaller proportions of patients in the tolvaptan cohorts had a history of hypertension compared with the control cohorts. Also carried through from the full analysis set, patients in the tolvaptan cohorts had a greater mean age at ADPKD diagnosis.

### Estimated Annual Rate of Change in eGFR

**Figure 2:**
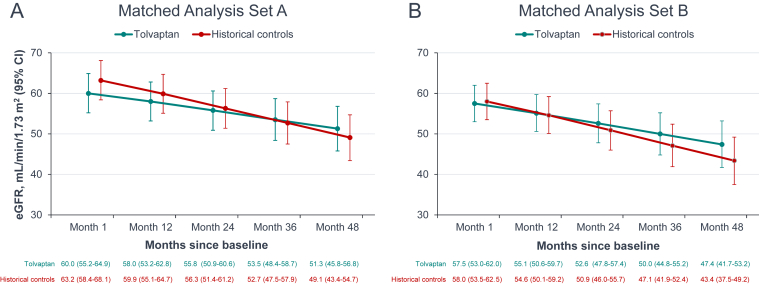
Estimated glomerular filtration rate over time as derived from the mixed model, matched analysis sets. CI, confidence interval; eGFR, estimated glomerular filtration rate.

Fig 2 shows eGFR over time, estimated from the mixed model for each analysis set. In set A, eGFR in the tolvaptan-treated cohort at 1 month was lower than in historical controls, which is due to the acute hemodynamic effect of tolvaptan (Fig 2A). At month 48, however, eGFR was lower in the control cohort than the tolvaptan cohort because of a steeper rate of decline among controls.

As shown in Fig 2B, eGFR at month 1 was similar between the tolvaptan and control cohorts, since month 1 eGFR served as the baseline eGFR value for set B, and the cohorts were matched for this variable. As in set A, eGFR in set B declined more rapidly to month 48 in the control cohort than in the tolvaptan cohort.

**Figure 3:**
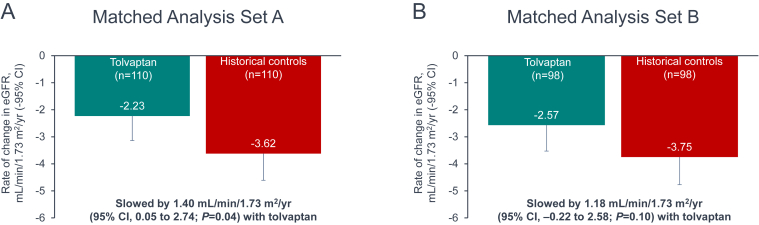
Annual change in estimated glomerular filtration rate as derived from the mixed model, matched analysis sets. CI, confidence interval; eGFR, estimated glomerular filtration rate; yr, year.

The annual rate of change in eGFR obtained from the mixed model was compared between the tolvaptan and control cohorts of each analysis set (Fig 3). In set A, there was a statistically significant slowing of the annual decline rate by 1.40 mL/min/1.73 m^2^ (95% confidence interval [CI], 0.05-2.74; *P* = 0.04) in patients receiving tolvaptan, when compared with historical controls. In set B, there was a trend of slowing in the annual eGFR decline rate by 1.18 mL/min/1.73 m^2^ (95% CI, −0.22 to 2.58; *P* = 0.10) in tolvaptan-treated patients versus historical controls.

To assess the potential effects of residual differences between the matched cohorts, a sensitivity analysis was conducted using the same mixed model methodology but including adjustment for the effects of race/ethnicity and history of hypertension. The treatment effect of tolvaptan for set A (1.40 mL/min/1.73 m^2^ per year [95% CI, 0.06-2.74; *P* = 0.04]) and set B (1.18 mL/min/1.73 m^2^ per year [95% CI, −0.22 to 2.57; *P* = 0.10]) in the adjusted analysis was nearly indistinguishable from that for set A and set B in the original analysis.

## Discussion

Results of this study provide insights on the effectiveness of tolvaptan within the context of regular nephrology practice in the United States for the treatment of patients with ADPKD who are at increased risk of rapid progression. Reductions in the rate of eGFR decline with tolvaptan were obtained from the comparisons of patients matched for baseline characteristics. The decrease with tolvaptan relative to controls was 1.40 mL/min/1.73 m^2^ per year for set A and 1.18 mL/min/1.73 m^2^ per year for set B, with directionally consistent results in both sets. The treatment effect in set B was of borderline statistical significance, with the difference in significance from set A potentially because of a smaller sample size.

The use of historical controls enabled comparison with patients not treated with tolvaptan, but this research design and the collection of data by survey entailed methodological limitations. Participating nephrologists were selected via convenience sampling, potentially limiting the generalizability of the results. Also, the selected patients may not be representative of the general population of US adults with ADPKD who are eligible to receive tolvaptan. In addition, patient data, such as diagnoses and laboratory measurements collected from medical records, may contain inaccuracies and may not be measured with the same method. A registry study or use of electronic medical records could provide prospective data to mitigate some of these limitations, provided that patients not treated with tolvaptan are available for comparison and that differences between tolvaptan-treated and non–tolvaptan-treated patients can be adjusted for.

Although historical controls were matched to cases on key patient characteristics, and the results of a sensitivity analysis further adjusted for imbalanced covariates were similar to those of the original matched analysis, the potential for residual confounding still exists.^17^ For example, Mayo imaging class was only available for the historical controls and not for tolvaptan-treated cases and therefore could not be controlled. Some cases in the full set were not matched with a control, which may further limit generalizability and reduce statistical power. Using historical controls could introduce non-contemporaneous bias, because clinical practice may have evolved over time. Finally, requiring treatment of at least 2 years for inclusion in the tolvaptan cohort allowed assessment of tolvaptan effect over multiple years of follow-up but did not generate an analysis set representative of the population starting therapy. A proportion of patients who start tolvaptan will discontinue because of aquaretic adverse effects, for example, 8.3% of participants in the tolvaptan arm of the TEMPO 3:4 trial.^1^ The REPRISE trial was designed to assess tolvaptan tolerability with regard to aquaretic adverse events during a 5-week, single-blind, tolvaptan titration and run-in period; 4.6% of participants discontinued for this reason during the single-blind phase, and 2.1% of the remaining study population randomized to the double-blind tolvaptan arm later discontinued because of aquaretic adverse events.^2^

A strength of this study was the ability to assess outcomes outside of the closely monitored context of clinical trials, which may enroll particularly motivated participants, and evaluate whether the benefit observed in controlled trials is also evident in clinical practice. The nephrologists surveyed were drawn from all geographic regions of the United States and all types of practice, whereas clinical trial investigators typically represent a limited number of study centers with a focused interest in ADPKD.

In summary, it is reassuring that the 1.18-1.40 mL/min/1.73 m^2^ per year slowing in eGFR decline with tolvaptan in our analysis is consistent with data from earlier randomized, controlled trials. In the TEMPO 3:4 and REPRISE trials, tolvaptan reduced the annual rate of eGFR decline by 1.20-1.27 mL/min/1.73 m^2^ versus placebo across populations with earlier to later-stage ADPKD.^2^ In conjunction with the findings of an earlier study of tolvaptan effectiveness in nephrology practice that compared eGFR decline before and after tolvaptan initiation,^6^ our research supports the effectiveness of tolvaptan in preserving kidney function in patients treated in routine clinical settings.
